# A two‐wave study on the effects of cognitive demands of flexible work on cognitive flexibility, work engagement and fatigue

**DOI:** 10.1111/apps.12392

**Published:** 2022-05-10

**Authors:** Lars Uhlig, Christian Korunka, Roman Prem, Bettina Kubicek

**Affiliations:** ^1^ Institute of Psychology University of Graz Graz Austria; ^2^ Faculty of Psychology University of Vienna Vienna Austria

**Keywords:** cognitive demands, engagement, flexible work, personal development, strain

## Abstract

Cognitive demands of flexible work are the specific cognitive demands of planning of working times, planning of working places, structuring of work tasks and coordinating with others that arise from flexible work organisation. Although these demands have become increasingly widespread, their consequences are not well understood. We propose that cognitive demands of flexible work are challenge stressors that can benefit employees, by adding to their cognitive flexibility and work engagement, but also impair employees by causing fatigue. Hypotheses were tested using a two‐wave study design in a sample that recently switched to a more flexible work organisation (*N* = 279). Data were analysed using structural equation modelling. We found that planning of working times and planning of working places were related to increases in cognitive flexibility, and coordinating with others was related to increases in work engagement. No significant relations with fatigue were found. Thus, the results suggest that cognitive demands of flexible work helped employees to personally develop and feel motivated at work. However, effects on work engagement were rather small. Future research should control potential confounding variables more thoroughly and examine effects on short‐term strain outcomes.

## INTRODUCTION

Today, intense competition and highly dynamic markets have forced organisations to implement flexible organisational structures in order to adapt to rapidly changing environmental demands. This trend is further promoted by technological developments such as information and communication technologies that both enable and drive organisations to become more flexible (Cascio & Montealegre, 2016; Rosa, 2013). For example, information and communication technologies allow for remote collaboration and therefore greater spatial flexibility. Moreover, they increase the speed of communication and the rate of change in markets, hence they also push organisations to implement more flexible organisational structures, which ultimately results in a higher flexibility for employees (Grant & Parker, 2009).

These changes have been largely recognised in the literature (Allvin et al., 2011; Cascio, 2003; Cascio & Montealegre, 2016), and a variety of studies have examined the effects of different aspects of flexible work such as the availability or use of flexible work arrangements (ter Hoeven & van Zoonen, 2015; Vander Elst et al., 2017) or employee autonomy (De Spiegelaere et al., 2016). However, as noted in a recent review on remote work, most studies on flexible work have not yet considered the cognitive dimensions of such work arrangements (Charalampous et al., 2019). This is an important omission, given that new demands stemming from flexible work are largely of a cognitive nature (Allvin et al., 2011). In a flexible work regime, employees are responsible for deciding and planning when, where, how and with whom they work^1^ (Allvin et al., 2011; Grant & Parker, 2009; Kubicek et al., 2017; Prem et al., 2021). They are expected to organise their work in a self‐reliant manner and in a way that they can perform effectively in a flexible and dynamic work environment (Allvin et al., 2011; Kubicek et al., 2015, 2017). As these new demands draw largely on cognitive operations such as planning, decision‐making and problem‐solving (Grant & Parker, 2009; Hertel et al., 2006; Schulze & Krumm, 2017; Väänänen et al., 2020), they can be described as cognitive demands of flexible work (Prem et al., 2021). Being a central aspect of flexible work, it is crucial to investigate these demands and their consequences to advance scholarly knowledge on flexible work.

Building on the challenge‐hindrance stressor framework (Crawford et al., 2010; LePine et al., 2005), we argue that cognitive demands of flexible work are challenge stressors. Challenge stressors are stressors that hold the potential to affect employees positively, for example, by contributing to their personal development or increasing their work engagement (Crawford et al., 2010; LePine et al., 2005). Both of these outcomes are likely for cognitive demands of flexible work. On the one hand, cognitive demands of flexible work could contribute to the personal development of employees by increasing their cognitive flexibility. Employees could learn to adapt to changing environments, consider and integrate others' perspectives, and flexibly solve problems (Dennis & Vander Wal, 2010; Kohn & Schooler, 1983). Various scholars have acknowledged that flexible work offers opportunities for such personal development (Allvin et al., 2011; Hertel et al., 2006; Wang & Haggerty, 2011), but there is still a lack of empirical studies on this matter. On the other hand, cognitive demands of flexible work could also increase employees' work engagement through their challenging and stimulating nature. Cognitive demands of flexible work require frequent adaptation to a dynamic and complex environment and can be mentally challenging (Allvin et al., 2011; Prem et al., 2021). Coping with these demands could give employees a sense of achievement and satisfy their need for competence and so increase their work engagement (Gagné & Deci, 2005; LePine et al., 2005; Ryan & Deci, 2017).

However, the gains of challenge stressors do not come without a cost (Crawford et al., 2010; LePine et al., 2005). The planning, structuring and coordinating that are linked to cognitive demands of flexible work are likely effortful (Frese & Zapf, 1994; Sjåstad & Baumeister, 2018) and could increase fatigue in employees (McEwen, 1998). Taking the positive (cognitive flexibility and work engagement) and negative (fatigue) aspects together, cognitive demands of flexible work would show ambivalent effects: On the one hand, they could allow employees to personally develop and foster their work engagement. On the other hand, they could cause employees to suffer from strain. So far, only few studies have investigated both beneficial and detrimental outcomes of flexible work in a single study (ten Brummelhuis et al., 2012; ter Hoeven & van Zoonen, 2015). Doing so is however important as it allows us to understand potential trade‐offs between outcomes (Parker et al., 2017).

Most studies on flexible work examining beneficial and detrimental outcomes so far used cross‐sectional designs (Charalampous et al., 2019), which have various limitations. Most importantly, as they measure all variables at the same time, they do not allow to examine changes in the variables of interest. In addition, cross‐sectional studies are more susceptible to methodological artefacts, such as common method bias (Podsakoff et al., 2003). Both of these limitations can be mitigated by using a two‐wave study design and so provide stronger indications for causal effects (Podsakoff et al., 2003, 2012). To address these issues, this study will investigate the effects of cognitive demands of flexible work on cognitive flexibility, engagement and fatigue using a longitudinal study design. We will investigate these effects in an organisational sample that has recently relocated to a new activity‐based flexible office. The organisational sample stems from the headquarter of an organisation in the logistics sector. With the sample having recently relocated to a new, activity‐based flexible office, we expect that the hypothesised effects will be strongest, as employees will not yet have developed routines in managing cognitive demands of flexible work. Further, as the sample stems from the headquarter of the organisation, participants in this study were working in a broad variety of departments with many occupations that can be found outside the logistics sector, such as marketing, HR, IT or accounting. Thus, our findings should be less confined to the logistics sector of the sample.

Our study adds to the literature in at least two ways. First, it will improve scholarly understanding of the cognitive demands of flexible work and their consequences. Shedding light on this issue is important as these demands are a central aspect characterising flexible work. Using a two‐wave study design, we answer Charalampous et al.'s (2019) call for more advanced research designs in examining the relationships between flexible work and employee outcomes. Second, we extend knowledge on the potential outcomes of flexible work by examining effects on cognitive flexibility. Increases in cognitive flexibility would represent a form of personal development that is an under‐researched, but highly relevant outcome of work, both for employees and organisations (Parker, 2014). Moreover, by examining the effects on cognitive flexibility, engagement and fatigue in parallel, our study may inform scholars and practitioners on potential trade‐offs between these outcomes.

## THEORETICAL BACKGROUND AND HYPOTHESES

### Defining cognitive demands of flexible work

Flexibility in organisations can be observed along the dimensions of time, place, performance and coordination (Allvin et al., 2011), meaning that employees are flexible in deciding when and where they work, how they perform their work, and with whom and how they collaborate. The flexibility along these four dimensions places specific cognitive demands on employees, with which employees have to deal in addition to their other demands (Prem et al., 2021). These cognitive demands of flexible work comprise planning of working times, planning of working places, structuring of work tasks and coordinating with others (Allvin et al., 2011; Grant & Parker, 2009; Kubicek et al., 2017; Prem et al., 2021).

Regarding employees' flexibility in deciding when to work, the introduction of flexible work schedules and collaboration across different time zones often requires organisation members to vary and adapt when they work, for how long they work and on which days they work (Höge & Hornung, 2015; Prem et al., 2021). Work hours have become more scattered throughout the day (Eurofound and the International Labour Office, 2017), and the task of managing these working hours has shifted from central management to the employees (Allvin et al., 2011). Employees must decide when to work in light of their work role, task deadlines, scheduled meetings and opportunities to collaborate with colleagues. Moreover, employees must incorporate time for their private needs, such as having time for family, friends, and leisure activities and being able to recover sufficiently between working days. This requires that employees plan their working time in terms of when they work, how long they work and when they take breaks.

Flexibility along the dimension of place can be observed in various ways in contemporary organisations. First, there is mobile work: This refers to employees working from home, in cafés and coworking spaces, on the road, or while with a client, typically using ICT (Eurofound and the International Labour Office, 2017). Second, higher flexibility can also be observed inside office buildings. Activity‐based office concepts, in which employees choose among different work areas depending on their work tasks, are becoming increasingly widespread (Wohlers & Hertel, 2017). The working places from which employees have to choose in mobile work and activity‐based offices tend to differ with regard to how well they fit certain tasks and employees' needs. For example, a quiet desk at home might be well suited for tasks requiring intense concentration, whereas a workplace in an open office might better fit tasks requiring frequent consultations with colleagues (Hoendervanger et al., 2021). As employees are responsible for planning and deciding where they work according to their tasks and needs, they face cognitive demands arising from planning their working place (Prem et al., 2021).

Flexibility in performance refers to the shift in organisations from predefined, structured tasks to a more decentralised management. This requires from employees to take an autonomous, self‐reliant approach to their work tasks and goals (Allvin et al., 2011; Griffin et al., 2007). As uncertainty and dynamics of change increase in the work environment, organisations rely on their employees to define their work tasks, make decisions about appropriate work methods, plan work steps ahead of time and monitor their work progress (Grant & Parker, 2009; Griffin et al., 2007). These demands can be summarised as cognitive demands of structuring work tasks (Prem et al., 2021).

Finally, higher organisational flexibility also involves demands related to coordinating with others. As many businesses and tasks are highly complex and require people with diverse abilities and knowledge, interdependence in organisations has increased substantially (Allvin & Movitz, 2017; Grant & Parker, 2009; Wegman et al., 2018). On the other hand, teams and projects are put together for the short term and often on short notice. In addition, the members of these teams often work flexibly, choosing independently when and where to work. With greater interdependence and fewer shared routines, employees must invest more time and effort in organising collaborations (e.g., scheduling meetings), finding a common approach to tasks and problems and exchanging information (Grant & Parker, 2009; Prem et al., 2021). Thus, employees in flexible work organisations also face cognitive demands related to coordinating with others.

### Consequences of cognitive demands of flexible work

We draw on the challenge‐hindrance stressor framework to develop and test a model of the consequences of cognitive demands of flexible work. The challenge‐hindrance stressor framework categorises work stressors into hindrance stressors and challenge stressors (Crawford et al., 2010; LePine et al., 2005; O'Brien & Beehr, 2019). Hindrance stressors are such stressors that obstruct employees in their goal achievement and are therefore mainly related to detrimental outcomes in employees (LePine et al., 2005). Challenge stressors, on the other hand, are thought to also benefit employees: Overcoming these stressors gives employees the opportunity to learn and gain a sense of accomplishment (LePine et al., 2005). Hence, they allow employees to personally develop and foster their engagement (Frese & Zapf, 1994; LePine et al., 2005). Nonetheless, because of their demanding nature, coping with challenge stressors requires effort, which can subsequently cause strain in employees (LePine et al., 2005). As we will argue, this should also apply for cognitive demands of flexible work.

### Cognitive flexibility

Cognitive flexibility refers to individuals' capabilities to be efficacious in a changing environment, consider different perspectives and being able to come up with solutions to new or difficult problems (Dennis & Vander Wal, 2010). The complexities of a flexible work environment give employees many possibilities to practice these skills. For example, when planning their working times and working places, employees will have to consider and integrate different demands stemming from their work tasks, their colleagues and supervisors, as well as their private life and personal needs. Similarly, being responsible for coordinating with others will require employees to engage in perspective‐taking. Further, as employees structure their work tasks in a dynamic environment, they could face unforeseen problems, which then require flexible problem‐solving (Parke et al., 2018). Constantly applying certain skills, competencies and behaviours at work may cause these skills to become automatised, form a general tendency in employees' behaviour and so increase employees' cognitive flexibility (Frese & Zapf, 1994; Zacher & Frese, 2018).

The idea that job demands can trigger such processes of personal development can be found in many influential theories of work design, including the job characteristics model (Hackman & Oldham, 1976), the job demand–control model (Karasek & Theorell, 1990) and action regulation theory (Frese & Zapf, 1994; Zacher & Frese, 2018), but only few studies have examined such effects. One of the first longitudinal studies on this topic was conducted by Kohn and Schooler (1983), who found positive effects of job complexity on individuals' cognitive flexibility. Evidence for such effects in the context of flexible work was reported by Nurmi and Hinds (2016). Participants in their study reported that collaborating with people from diverse backgrounds taught them new perspectives and the challenging nature of the work required them constantly to learn how to tackle new problems (Nurmi & Hinds, 2016). Thus, we expect that facing cognitive demands of flexible work will increase the cognitive flexibility of employees over time.

Hypothesis 1 Cognitive demands of flexible work are positively associated with increases in cognitive flexibility.

### Work engagement

Being engaged at work can be described as a state of vigour, dedication and absorption (Schaufeli & Bakker, 2004). This means that employees are motivated to invest effort, feel enthusiastic about their work and can be immersed in their work (Bakker, 2011). The results of the meta‐analysis of Crawford et al. (2010) on the challenge‐hindrance stressor framework show that an intellectually stimulating work, as for example represented by high job complexity, is a strong predictor of work engagement. Intellectually stimulating work can be a motivator in itself as it makes work more interesting (Hackman & Oldham, 1976), but it also allows employees to satisfy their need for competence (Gagné & Deci, 2005; van den Broeck et al., 2016). The need for competence refers to the basic need for experiences of competence, mastery and accomplishment, which is often a central motive for individuals to engage in activities and invest effort (Ryan & Deci, 2017). Planning, structuring and coordinating work self‐reliantly and adapting flexibly to changing work conditions, activities necessary for coping with cognitive demands of flexible work, should also be intellectually stimulating and could therefore increase work engagement in employees. In line with this argument, Prem et al. (2021) found positive relationships between cognitive demands of flexible work and work engagement. Thus, we expect that cognitive demands of flexible work increase work engagement in employees over time.

Hypothesis 2 Cognitive demands of flexible work are positively associated with increases in work engagement.

### Fatigue

Besides their beneficial effects, challenge stressors should also be demanding and coping with them should be effortful (LePine et al., 2005). This also applies for cognitive demands of flexible work. Engaging with these demands involves the cognitive operations of planning, decision‐making, organising and problem‐solving (Hertel et al., 2006; Parke et al., 2018; Schulze & Krumm, 2017). Working flexibly means operating in a changing environment with low levels of structure (Allvin et al., 2011; Parke et al., 2018). Both together makes it necessary for employees to constantly analyse their environment and its demands and to adapt their plans, decisions and procedures accordingly (Bäcklander et al., 2018; Jett & George, 2003; Parke et al., 2018). Doing so requires conscious attention and effort and could result in fatigue in employees (Frese & Zapf, 1994; Sjåstad & Baumeister, 2018; Zacher & Frese, 2018). In line with this argument, various studies have found that demands resulting from flexible work can exhaust employees (Bäcklander et al., 2018; Höge & Hornung, 2015; Pérez‐Zapata et al., 2016; Schmitt et al., 2012; Väänänen et al., 2020). Moreover, repeated or prolonged elevated levels of fatigue are assumed to lead to stabilised symptoms of exhaustion (Geurts & Sonnentag, 2006; McEwen, 1998; Hockey, 2013). Chronically high levels of cognitive demands of flexible work may therefore lead to long‐term accumulation of fatigue. Hence, we expect that cognitive demands of flexible work are positively associated with increases in fatigue over time.

Hypothesis 3 Cognitive demands of flexible work are positively associated with increases in fatigue.

## METHOD

### Participants and procedure

All participants provided informed consent prior to participating in the study. They participated voluntary and anonymously and were free to withdraw from the study at any point. As we followed standard procedures in applied psychological research and did not touch on sensitive topics, the procedure and the materials of the study were not reviewed by an ethics committee. Concerning the ethical standards for research, the protocol fully complied with the ethical principles of psychologists and the code of conduct of the American Psychological Association (2017).

Data were collected from employees working at the headquarters of a large company in the logistics sector. The employees worked in various departments responsible for the main business activities of the company, including strategy, communication, human resources and IT. Their tasks included administrative, professional and managerial work. Participants had to work full time (at least 35 h/week) to be included in the study.

The study was conducted in the context of a scientific evaluation of a relocation of the organisation's headquarter. With the relocation, a new office design was implemented. Previously, the office had been designed with fixed desks in small to medium‐sized conventional offices shared by two to three people or small open office spaces. At the new location, a flexible office with desk sharing was implemented. Here, employees had to choose their desk each day or multiple times per day depending on their task and the colleagues with whom they needed to coordinate. The office design was intended to foster and facilitate cooperation between employees and their colleagues within and across teams. Thus, the participants in this study were expected to face a recent increase in cognitive demands of flexible work. Such circumstances are well suited to examining our hypotheses, as increases in cognitive flexibility, engagement and fatigue should be highest immediately after an increase in workplace flexibility and before adaptation processes have taken place. When planning the timing of the first measurement, we consulted with the organisation to find a time point that was as close as possible to the relocation, but after most disorganisation from the process of relocating was resolved. The data used in this study were collected in the surveys conducted 2 (T1) and 6 months (T2) after the relocation.^2^ Thus, the time lag between the two measurement points was 4 months. Time lags should be chosen based on the stability of the constructs and considering enough time for the effects of interest to unfold (Dormann & Griffin, 2015). Cognitive flexibility, engagement and fatigue should be relatively stable constructs (Dennis & Vander Wal, 2010; Garst et al., 2000; Gillet et al., 2019: Seppälä et al., 2009) and the underlying mechanisms learning, achievement and exhaustion of resources, that we expect to drive the changes in these constructs, require repeated exposure to the stressors. Thus, we chose a time lag of 4 months that is on the upper end regarding the recommendation by Dormann and Griffin (2015) to conduct ‘shortitudinal’ studies, but still substantially shorter than most time lags in studies examining similar processes (Guthier et al., 2020; Lesener et al., 2018; Li et al., 2014). According to talks with the organisation and people on the ground most disorganisation from the process of the relocation was resolved at T1.

Employees were contacted and invited to participate in the online survey by company representatives. The invitation to the survey explained the research purpose and emphasised that participation was voluntary and anonymous. At each measurement point, the survey was sent to all employees working at the headquarters (approximately 1000 at the time). Of these, 501 filled out the survey at T1 and 438 at T2. The final sample comprised of 660 participants, of which 279 participants had filled out the survey at both T1 and T2. The majority of participants were male (59.0%), average age was 44.01 years (*SD* = 9.74 years) and average job tenure was 14.06 years (*SD* = 13.57 years). Over 48.2 per cent of participants had a degree from a university, 31.8 per cent had a high school degree and 15.5 per cent had finished an apprenticeship or had received secondary education.

We tested for differences in sample characteristics between respondents who participated only at T1 and those who participated at both T1 and T2. We found no significant differences in relevant variables. We concluded that participants who dropped out between T1 and T2 did not differ meaningfully from the final sample and that study attrition was not a concern. Details on the analysis and the results can be found in the supporting information.

### Measures

For all measures at T1, participants were asked to refer in their answers to the preceding 2 months (the time after the relocation). At T2, participants were asked to refer in their answers to the preceding 4 months (i.e., the time between T1 and T2). By asking participants about a shorter timeframe rather than their general experiences at work, we aimed to reduce bias from earlier experiences in their work careers and assess more accurately short‐term change processes in the timeframe of our study. Further, by asking for experiences during specific timeframes, we avoided that participants referred in their answers at both time points to the same or an overlapping set of experiences (Dormann & Griffin, 2015).

*Cognitive demands of flexible work* were measured at T1 with the scale developed by Prem et al. (2021), which contains four subscales measuring planning of working times, planning of working places, structuring of work tasks and coordinating with others. Participants responded to each item on a 5‐point Likert scale ranging from 1 (‘does not apply at all’) to 5 (‘fully applies’). Each subscale comprised three items. Example items are ‘Due to my flexible schedule, I had to decide how long I work on which weekdays’ (planning of working times, *α* = .77), ‘At work, I had to plan where to work on certain tasks, because I do not have the same work materials available everywhere’ (planning of working places, *α* = .83), ‘My job requires me to determine the sequence of my work steps on my own’ (structuring of work tasks, *α* = .92) and ‘My job often required me to come to an agreement with other people regarding a common approach’ (coordinating with others, *α* = .83).

*Cognitive flexibility* was measured with a shortened version of Dennis and Vander Wal (2010) scale. We used four items, which asked whether participants had considered different perspectives and approaches when facing difficult situations or interacting with other people. Participants responded to each item on a 5‐point Likert scale ranging from 1 (‘does not apply at all’) to 5 (‘fully applies’). A sample item is ‘I often looked at a situation from different viewpoints’. Cronbach's alphas for the subscale were .85 at T1 and .87 at T2.

*Work engagement* was measured with an ultra‐short version of the Utrecht Work Engagement Scale, which was also used by Eurofound (2017) in the 6th European Working Conditions Survey (Schaufeli, 2017). The scale was very similar to the UWES‐3 (Schaufeli et al., 2017), which was published after planning this study. Like the UWES‐3, the scale used in this study had one item each to measure the dimensions vigour, dedication and absorption (Schaufeli et al., 2017). However, the item to measure absorption was different than in the UWES‐3: Instead of using the item ‘I am immersed in my work’, we used the item ‘Time flies when I was working’. As Schaufeli et al. (2017) notes, both items correlate highly. The two other items were the same as in the UWES‐3. Participants responded to each item on a 5‐point Likert scale ranging from 1 (‘does not apply at all’) to 5 (‘fully applies’). A sample item of the scale is ‘I felt bursting with energy at my work’. Cronbach's alphas for the scale were .83 at T1 and .82 at T2.

Fatigue was measured with four items from the Profile of Mood Scales (McNair et al., 1971; German translation by Sonnentag et al., 2008). Participants indicated how often they had felt ‘fatigued’, ‘tired’, ‘spent’ and ‘exhausted’ after a day of work. Answers were given on a 5‐point Likert scale ranging from 1 (‘never’) to 5 (‘always’). Cronbach's alphas for the subscale were .90 at T1 and .91 at T2.

### Analytic strategy

**FIGURE 1:**
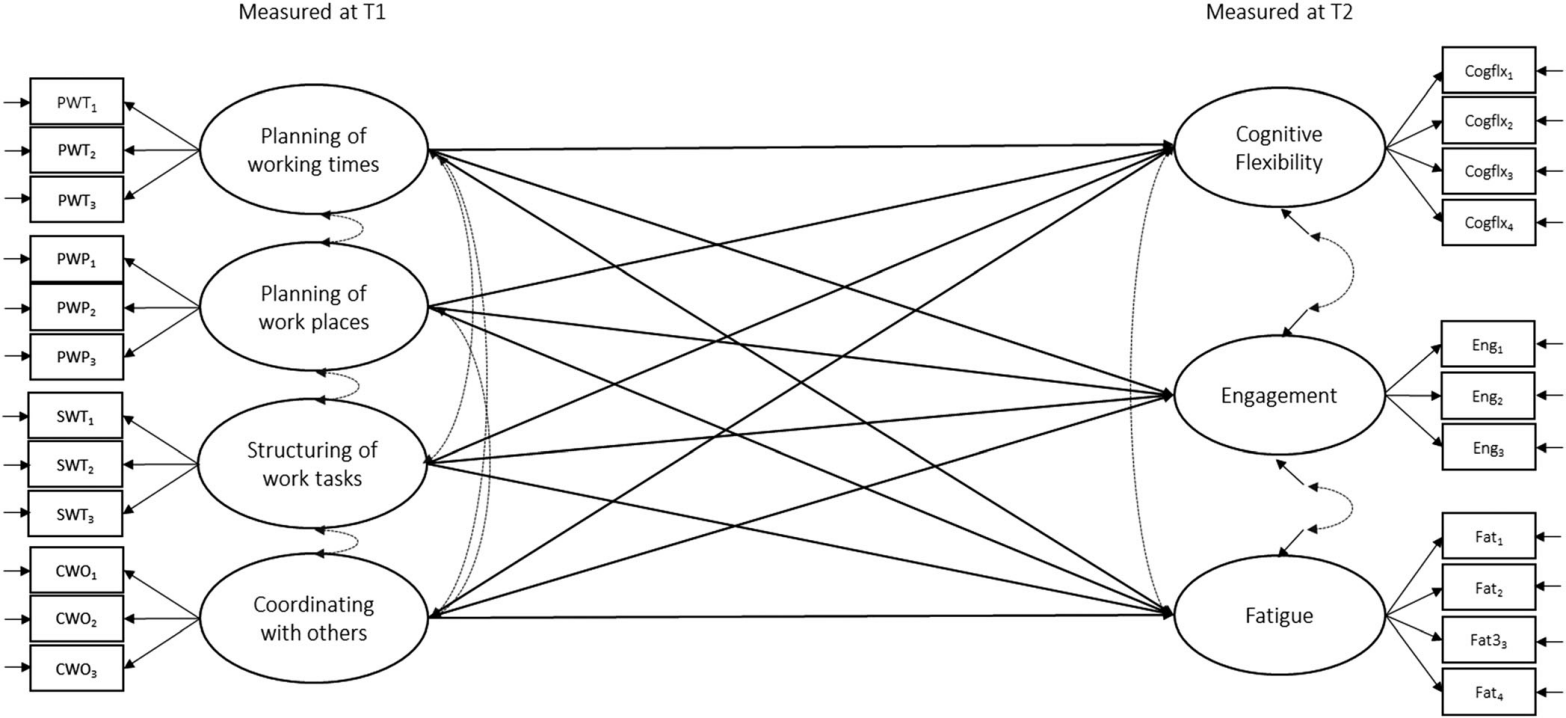
Structural equation model of the relationships of the four cognitive demands of flexible work with cognitive flexibility, engagement, and fatigue. *Note*: Cognitive flexibility, engagement and fatigue were controlled for their respective T1 measurements

We used structural equation modelling (SEM) to test the hypotheses that the four cognitive demands of flexible work, planning of working times, planning of working places, structuring of work tasks and coordinating with others, positively predict changes in cognitive flexibility, work engagement and fatigue. A graphical depiction of the specified model can be found in Figure 1. All measured constructs were specified as latent factors, resulting in a model with 10 latent factors in total. Cognitive flexibility, work engagement and fatigue at T2 were regressed on the four cognitive demands of flexible work, as well as on their respective measures at T1. For all variables that were measured at the same time point, covariances were specified, leading to a just‐identified structural model. Based on the recommendations from Cole and Maxwell (2003), residual covariances were specified for indicators of cognitive flexibility, work engagement and fatigue at T1 with their corresponding indicators at T2 to account for shared method variance.

All analyses were conducted using Mplus 8.5 (Muthén & Muthén, 1998–2017) and a maximum likelihood estimation with robust standard errors. To assess model fit, we considered various parameters, including chi‐square goodness of fit statistic (χ^2^), comparative‐fit indices (CFI), Tucker–Lewis index (TLI) and root mean square error of approximation (RMSEA), using the suggested cut‐off criteria by Hu and Bentler (1999). Because all hypotheses were directional, one‐tailed testing was used. As the effects of cognitive demands of flexible work could be confounded by job autonomy, we also executed an additional analysis in which we controlled for the effects of job autonomy. Details of the analysis and the results can be found in the supporting information.

## RESULTS

Table 1 presents the means, standard deviations and intercorrelations of all study variables. Cognitive flexibility, work engagement and fatigue showed a high stability in the mean levels from T1 to T2, as well as high autocorrelations. We first specified and tested a model that only included the stability paths of the dependent variables and in which the effects of all four cognitive demands of flexible work on the three dependent variables were fixed to zero. In the final model, we would then include the effects of the four cognitive demands of flexible work to examine if this improves model fit and contributes to explained variance in the dependent variables. Improvement in model fit was assessed using chi‐square difference test, as well as the Akaike information criteria (AIC), for which lower values indicate a better model fit.

**TABLE 1:** Intercorrelations of all variables

	1	2	3	4	5	6	7	8	9	10	11	12	13	14
1. Planning of working times (PWT)	—													
2. Planning of working places (PWP)	**.15**	—												
3. Coordinating with others (CO)	**.24**	**.33**	—											
4. Structuring of work tasks (ST)	**.31**	**.15**	.23	—										
5. Cognitive flexibility T1	**.16**	**.20**	**.35**	**.33**	—									
6. Engagement T1	**.20**	−.04	**.22**	**.31**	**.18**	—								
7. Fatigue T1	**−.18**	**.18**	−.06	−.10	−.01	**−.53**	—							
8. Cognitive flexibility T2	**.25**	**.26**	**.19**	.14	**.72**	**.24**	−.04	—						
9. Engagement T2	**.21**	−.06	**.25**	**.23**	**.26**	**.88**	**−.51**	.**33**	—					
10. Fatigue T2	**−.19**	.15	−.11	**−.14**	−.06	**−.42**	**.72**	−.07	**−.56**	—				
11. Gender	.02	.01	−.03	−.05	−.06	**.09**	.07	.01	.09	**.08**	—			
12. Age	−.09	**−.13**	**−.33**	.07	−.08	**−.09**	.01	−.09	−.03	**−.03**	−**.17**	—		
13. Tenure	−.09	**−.19**	**−.36**	−.04	**−.21**	−.08	.01	**−.19**	−.07	**.06**	**−.09**	**.74**	—	
14. Education	**.18**	**.17**	**.36**	.09	**.26**	.05	.01	**.20**	.07	**.02**	**.08**	**−.41**	**−.55**	—
*M*	3.75	2.73	3.71	4.12	4.08	3.66	2.96	4.11	3.69	2.98	1.41	44.01	14.06	4.2
*SD*	0.96	1.11	0.85	0.75	0.58	0.75	0.85	0.58	0.71	0.83	0.49	9.74	13.57	1.07

*Note*: For bold values, *p* < .05.

The model that only included the stability paths showed a good fit (*χ* ^2^ = 744.319, df = 485, CFI = .969, TLI = .964, RMSEA = .028, AIC = 33,689.83). For all three dependent variables, measurements at T1 strongly predicted measurements at T2, that is, cognitive flexibility (*β* = .68, *p* < .001), work engagement (*β* = .84, *p* < .001) and fatigue (*β* = .69, *p* < .001). Measurements at T1 explained large amounts of variance in measurements at T2, that is, cognitive flexibility (*R* ^2^ = .468, *p* < .001), work engagement (*R* ^2^ = .709, *p* < .001) and fatigue (*R* ^2^ = .477, *p* < .001). These results indicate a high stability in all three constructs over the two measurements.

The final model in which the effects of the cognitive demands of flexible work were freely estimated showed a good fit (*χ* ^2^ = 714.23, df = 473, CFI = .971, TLI = .966, RMSEA = .028, AIC = 33,680.83). Compared with the model that only included the stability paths, the final model resulted in an improvement in model fit (Δ*χ* ^2^ = 30.089; Δdf = 12; *p =* .003; ΔAIC = −9.00). Hypothesis 1 stated that cognitive demands of flexible work will be positively related to increases in cognitive flexibility. We found significant positive relationships of planning of working times (*β* = .20, *p* = .001), and planning of working places (*β* = .15, *p* = .008) with cognitive flexibility, and no positive relationships for structuring of work tasks (*β* = −.15, *p* = .990) and coordinating with others (*β* = −.13, *p* = .973). The results therefore showed that higher demands of planning of working times and planning of working place were related to increases in cognitive flexibility at T2, but no such relationship was found for structuring of work tasks and coordinating with others. Compared to the model that only included the stability paths, the final model resulted in an increase of 7.2 per cent in explained variance in cognitive flexibility at T2. Hypothesis 1 was partly supported.

Due to the negative estimates for the relationship between structuring of work tasks and coordinating with others with cognitive flexibility, we did an exploratory analysis in which we examined these relationships using two‐tailed testing. This analysis revealed a negative relationship for structuring of work tasks (*β* = −.15, *p* = .021) and a non‐significant relationship for coordinating with others (*β* = −.13, *p* = .054).

Hypothesis 2 stated that cognitive demands of flexible work will be positively associated with increases in work engagement. We found no significant relationships for planning of working times (*β* = .04, *p* = .759), planning of working places (*β* = −.05, *p* = .824) and structuring of work tasks (*β* = −.06, *p* = .852). Coordinating with others showed a significant positive relationship with work engagement (*β* = .10, *p* = .049). Our results therefore showed that higher demands to coordinate with others was positively related to increases in work engagement, but none of the other cognitive demands of flexible work showed such a relationship. Compared with the model that only included the stability paths, the final model resulted in an increase of 0.7 per cent in explained variance in work engagement at T2. Hypothesis 2 was partly supported.

Hypothesis 3 stated that cognitive demands of flexible work are positively associated with increases in fatigue. We found no significant effects for planning of working times (*β* = −.05, *p* = .812), planning of working places (*β* = .07, *p* = .875), structuring of work task (*β* = −.06, *p* = .876) or coordinating with others (*β* = −.06, *p* = .846) on fatigue. Compared with the model that only included the stability paths, the final model resulted in an increase of 1.7 per cent in explained variance in fatigue at T2. Hypothesis 3 had to be rejected (Table 2).

**TABLE 2:** Results of the structural equation modelling

	Cognitive flexibility		Engagement		Fatigue	
	Estimate	*SE*	Estimate	*SE*	Estimate	*SE*
Planning of working times	.20**	.07	.04	.05	−.05	.05
Planning of working places	.15**	.06	−.05	.06	.07	.06
Structuring of work tasks	−.15	.07	−.06	.06	−.06	.05
Coordinating with others	−.13	.07	.10*	.06	−.06	.05
Cognitive flexibility (T1)	.72***	.06				
Engagement (T1)			.83***	.05		
Fatigue (T1)					.66***	.04
*R* ^*2*^	.540***		.716***		.494***	

* *p* < .05.

** *p* < .01.

*** *p* < .001.

## DISCUSSION

To summarise, we found that planning of working times and planning of working places but not structuring of work tasks and coordinating with others positively predicted increases in cognitive flexibility. Regarding work engagement, we found that only coordinating with others predicted increases in work engagement, but none of the other cognitive demands of flexible work. No positive effects for any of the cognitive demands of flexible work on fatigue were found. Thus, cognitive demands of flexible work in part showed relationships with beneficial outcomes, but showed no relationships with detrimental outcomes.

### Theoretical implications

Our study shows that cognitive demands of flexible work are a relevant aspect of flexible work arrangements (Allvin et al., 2011; Charalampous et al., 2019). Working flexibly is characterised by specific cognitive demands to plan, structure and coordinate work along the dimensions of time, place, performance and coordination (Allvin et al., 2011; Prem et al., 2021). Cognitive demands of flexible work proved to predict increases in cognitive flexibility and engagement, two outcomes that are both relevant for employees and organisations.

Our results suggest that flexible work could contribute to the personal development of employees as they were positively related to increased cognitive flexibility. However, an interesting pattern appeared in the results: Only planning of working times and planning of working places were related to increases in cognitive flexibility, but no such effects were found for structuring of work tasks or coordinating with others. Whereas structuring of work tasks and coordinating with others are demands that only relate to the work sphere, planning of working times and planning of working places are often related to managing demands stemming from different spheres of life, such as leisure time and work. For example, employees might adapt their working times flexibly in order to run private errands, or they may decide in a busy week to work from home allowing them to have lunch with their family. This means that employees with high demands to plan working times and working places probably switch more often between different life spheres during a day. To avoid and resolve conflicts between spheres, they will also have to consider different perspectives when planning their working times and working places (de Janasz & Behson, 2007; Parker et al., 2008). Both, switching often between different life spheres and perspective‐taking could be beneficial for cognitive flexibility. Further, as both demands involve life spheres outside of work, learning processes may be less limited to work‐related skills and also influence employees' behaviour outside of work (Frese & Zapf, 1994). As cognitive flexibility is not specifically related to the work sphere, this could make effects on cognitive flexibility more likely. Structuring of work tasks and coordinating with others, on the other hand, are only related to the work sphere and learning processes linked to these demands may therefore be less likely to affect cognitive flexibility. Even more, the results of an exploratory analysis suggest that structuring of work tasks could be negatively related to cognitive flexibility. Research on convergent and divergent thinking has shown that switching often between different tasks can benefit the cognitive flexibility of employees (Lu et al., 2017). Following from this, high demands to structure work tasks could harm cognitive flexibility as high structuring could be linked to less task switching. In addition to these theoretical explanations, methodological issues could have also been relevant. Participants in our sample scored very high on structuring of work tasks. Thus, range restriction could have reduced the power to find an effect or biased the effect (Rousseau & Fried, 2001). We discuss this issue in more detail in Section 5.3.

Activity‐based offices are often implemented hoping to improve collaboration and save costs (Wohlers & Hertel, 2017). Whereas the particular consequences of such an implementation seem to depend on the execution of the implementation and the design of the new office (Bergsten et al., 2021; Gerdenitsch et al., 2018), activity‐based offices often fall short of the expected benefits (Kim & De Dear, 2013; Haapakangas et al., 2019). Instead, disadvantages regarding distractions or loss of privacy seem to prevail (Kim & De Dear, 2013; Hodzic et al., 2021). Our findings, however, hint at a potential pathway via which activity‐based offices could still benefit employees: by increasing cognitive demands of flexible work, they could subsequently also benefit the cognitive flexibility of employees. However, having no data on cognitive demands of flexible work before the relocation, we cannot draw any conclusion on this matter. Instead, we want to encourage future research to examine this empirically.

Regarding work engagement, we found that only coordinating with others was related to increases in work engagement over time. High demands to coordinate with others could affect employees work engagement positively as they can be a constant source of stimulation, both intellectually and socially. This should hold especially true in a dynamic work environment, in which employees have multiple team memberships and teams also change often. Coordinating with others often involves collaborating with professionals and experts who have different skills and knowledge meaning that employees can profit from new information and knowledge sharing (van de Brake et al., 2018). And as employees bring their own unique skills and knowledge to the collaboration, they could satisfy their need for competence. Besides satisfying the need for competence, working together in a team or project could also allow employees to satisfy their need for relatedness (Ryan & Deci, 2017; van den Broeck et al., 2016).

However, it is important to note that the found relationship with work engagement should be interpreted with caution. The additional variance explained of cognitive demands of flexible work beyond the autoregressive effect of work engagement was only 0.7 per cent. Further, as can be seen in the supporting information, the relationship was no longer significant after including job autonomy as a control variable. A potential explanation for this could be the very high stability of work engagement in our sample of *r* = .88 between T1 and T2. This left little additional variance to be explained and likely reduced the power to find a substantial effect (Adachi & Willoughby, 2015). Nonetheless, one should consider that the effect of coordinating with others on changes in work engagement could be rather small or non‐existent. Future research, preferably in samples with a lower stability in work engagement, is necessary to clarify this.

Considering the missing link with a strain outcome our study revealed an interesting pattern of relationships for cognitive demands of flexible work. Apparently, cognitive demands of flexible work seem to stimulate employees, but without putting a toll on them as challenge stressors are believed to do (LePine et al., 2005). Although it could be tempting to then categorise cognitive demands of flexible work as a resource, there still remain important differences. Most importantly, resources should be positively related to well‐being (Van den Broeck et al., 2010). Further, they should be functional in coping (Bakker & Demerouti, 2007; Van den Broeck et al., 2010). In this regard, cognitive demands of flexible work still share more characteristics with a challenge stressor, as they should require problem‐focused coping (LePine et al., 2005; Prem et al., 2021; Van den Broeck et al., 2010). Interestingly, our study is not the first to show that some cognitive demands might not be related to strain (Meyer & Hünefeld, 2018; Van den Broeck et al., 2010). As Van den Broeck et al. (2010) suggest, it could be that certain challenge stressors require some expenditure of effort, but without leading to strong and stable exhaustion. Such a differentiation between short‐term and long‐term exhaustion caused by challenge stressors would represent an important refinement of the challenge‐hindrance stressor framework. To move forward in this matter, we suggest that future research examines whether cognitive demands of flexible work cause more short‐term forms of fatigue, for example, by using diary studies.

### Practical implications

Our results add to the notion that flexible work arrangements can offer many advantages for employees and organisations. If employees plan their working times and working places self‐reliantly, this may not only help them to improve their work‐life balance, as earlier studies have already shown (Allen et al., 2013; Casey & Grzywacz, 2008; Hill et al., 2001), but could also contribute to their personal development. To foster such effects, organisations should offer employees flexibility regarding their working times, but also regarding the place from which they work, for example by implementing flexible trust‐based working hours, remote work or an activity‐based flexible office design. Further, our results suggest that organisations should put the task of coordinating with others in the hand of the employees as this could have motivational effects. Matrix and network organisations, flat hierarchies and a project‐based structure of the organisation could be suitable frameworks for this (Grant & Parker, 2009).

### Limitations

Various limitations of this study should be mentioned. First, we did not measure cognitive demands of flexible work before the relocation. Although it is a plausible explanation that the observed changes in the outcomes variables result from changes in cognitive demands of flexible work due to the relocation, our results only inform us on the influence of the level of cognitive demands of flexible work at T1. Thus, we suggest that future research examines how changes in cognitive demands of flexible work affect outcomes in employees.

Second, we did not control for general cognitive demands. Cognitive demands of flexible work likely correlate with general cognitive demands, and some of the here described effects could also play a role for general cognitive demands. However, in the initial validation of the cognitive demands of flexible work scale by Prem et al. (2021), the authors found evidence for divergent validity of cognitive demands of flexible work to other cognitive demands such as problem‐solving or information processing. Nevertheless, future research should control for general level of cognitive demands and investigate whether cognitive demands of flexible work explain additional variance in the outcomes.

Third, all measures were self‐reported, meaning that common method variance could have biased the results (Podsakoff et al., 2003, 2012). However, this was probably diminished by using a time lag between independent and dependent variables (Podsakoff et al., 2003, 2012). Nonetheless, future research should assess cognitive demands of flexible work using objective measures to verify our results. Variability in working times and working places, expert or supervisor ratings of structuring of work tasks, and the number of multiple team memberships could serve as objective indicators for cognitive demands of flexible work.

Fourth, our results allow for no causal interpretation of the results. Although a two‐wave study design was an important step forward from earlier cross‐sectional studies, it does not allow us to rule out that third variables confounded the effects. To do so, a field‐ or quasi‐experimental design would be necessary.

Finally, range restrictions could have biased the results. Participants in our sample scored very high on structuring of work tasks (*M* = 4.12 on a 5‐point Likert scale), and the demand showed a left‐skewed distribution. Such a range restriction in an independent variable reduces the power to find effects and may bias estimates strongly (Rousseau & Fried, 2001). Moreover, range restriction could have been aggravated as our sample stemmed from a single organisation and showed a high educational level with more than 48 per cent of the participants having at least a university degree (Rousseau & Fried, 2001). Future research should draw on more diverse samples, preferably recruiting participants from various organisations and sectors to reduce bias through range restriction.

### Conclusion

Rising flexibility in the world of work has given employees more freedom, but it has also put new demands on employees. These demands are largely of a cognitive nature as they consist of planning, structuring and coordinating along the dimensions of time, place, performance and coordination. Using a two‐wave study design, we examined whether these cognitive demands of flexible work can be understood as challenge stressor, which are related to both beneficial (cognitive flexibility and work engagement) and detrimental (fatigue) outcomes. We found that cognitive demands of flexible work were only associated with beneficial outcomes: Planning of working time and planning of working places were related to increases in cognitive flexibility, suggesting that flexible work environments can be beneficial for the personal development of employees. Coordinating with others were positively related to increases in work engagement, further underlining the motivational potential of flexible work arrangements. As none of the cognitive demands of flexible work were related to increases in fatigue, we found no evidence that these demands act as stressors. We suggest that future research controls for confounding variables, such as general levels of cognitive demands and common method variance, and examines whether cognitive demands of flexible work lead to strain on shorter time scales.

## CONFLICT OF INTEREST

The authors report no potential conflict of interest.

## ETHICS STATEMENT

The authors did not obtain formal ethical committee statements for this study, as the research did not touch on sensitive topics and the procedures were deemed non‐invasive.

## Supporting information


**Table S1** Data transparency table.
**Table S2** Results of the Structural Equation Modeling when Controlling for Job Autonomy

## Data Availability

The data used in this research are not readily available, as the participants have not been asked for consent to share the data publicly. Requests to gain access to the data should be directed to the corresponding author.

## ENDNOTES

^1^
It is important to differentiate between employee‐ and employer‐oriented flexibility as both refer to vastly different forms in the organisation of work and have different consequences (Uglanova & Dettmers, 2018). In this study, we solely refer to the former.

^2^
The scientific evaluation consisted of four online surveys, conducted 2 months before the relocation and 2, 6 and 12 months after the relocation. Data from this project were reported in two earlier studies (Hodzic et al., 2021; Prem et al., 2021). Both studies differed substantially from the present study in their research focus and hypotheses. There was no overlap with the data of the dependent variables or the relationships examined here. Detailed descriptions of the studies and their variables can be found in the data transparency table in the supporting information.
