# Supplementary material for: A two‐wave study on the effects of cognitive demands of flexible work on cognitive flexibility, work engagement and fatigue
Source: Appl Psychol. 2022 May 10;72(2):625–46. doi: 10.1111/apps.12392 (PMC10953014; doi:10.1111/apps.12392)
Supplement: Supplementary file 1 — Table S1 Data transparency table. Table S2 Results of the Structural Equation Modeling when Controlling for Job Autonomy [file APPS-72-625-s001.docx]

# Appendix

To examine whether study attrition was a concern, we tested whether participants who participated only at T1 and those who participated at both time points different significantly with regard to relevant variables. We found no significant differences in age, *t*(490) = 1.37, *p* = .17, gender, χ²(1, *N* = 501) = 1.71, *p* = .19, tenure, *t*(495) = 0.49, *p* = 0.62, leadership position, χ²(1, *N* = 499) = 0.003, *p* = .96, or level of education, χ²(4, *N* = 501) = 2.64, *p* = .62 between both samples. Further, there were no significant differences in the levels of planning of working times, *t*(496) = -2.83, *p* = .78, planning of working places, *t*(498) = -0.40, *p* = .69, structuring of work tasks, *t*(499) = -1.18, *p* = .24, or coordinating with others, *t*(498) = -1.39, *p* = .17. Further, both groups did not differ significantly with regard to cognitive flexibility, *t*(499) = -0.97, *p* = .33, work engagement, *t*(499) = -0.74, *p* = .46, or fatigue, *t*(498) = -0.94, *p* = .35, at time point 1.

We also ran an additional analysis in which we controlled for job autonomy. Job autonomy was measured at T1 with three items from the job control subscale of the Instrument for Stress-oriented Job Analysis (Semmer et al., 1999). Responses were given on a 5-point Likert scale ranging from 1 (“very little”) to 5 (“a lot”). A sample item is “Can you determine by yourself how to carry out your work?”. Cronbach’s alpha for the subscale was .87.

To control for job autonomy, we specified the same model as in Figure 1 and included job autonomy as a latent factor predicting cognitive flexibility, work engagement, and fatigue at T2. The model had a good fit (χ^2^ = 844.60, df = 565, CFI = .97, TLI = .965, RMSEA = .027, AIC = 36671.54). A summary of the results can be found in Table 3. The effects of the cognitive demands of flexible work on cognitive flexibility remained mostly unchanged after controlling for job autonomy. The effect of coordinating with others on work engagement was no longer significant (β = 0.08, *p* = .062).

| **Table 3.**  *Results of the Structural Equation Modeling when Controlling for Job Autonomy* | | | | | | |
| --- | --- | --- | --- | --- | --- | --- |
|  | Cognitive flexibility | | Engagement | | Fatigue | |
|  | Estimate | *SE* | Estimate | *SE* | Estimate | *SE* |
| Planning of working times | .20** | .07 | .04 | .05 | -.05 | .05 |
| Planning of working places | .15** | .06 | -.05 | .06 | .07 | .06 |
| Structuring of work tasks | -.17 | .08 | -.10 | .07 | -.03 | .06 |
| Coordinating with others | -.13 | .07 | .09 | .06 | -.06 | .05 |
| Job Autonomy (T1) | .03 | .09 | .03 | .09 | -.05 | .07 |
| Cognitive flexibility (T1) | .72*** | .06 |  |  |  |  |
| Engagement (T1) |  |  | .81*** | .05 |  |  |
| Fatigue (T1) |  |  |  |  | .66*** | .04 |
| *R²* | .54*** | | .72*** | | .50*** | |
| *Note*. **p < .05; ** p < .01; *** p < .001* | | | | | | |
